# Effect of Abdominal Circumference on the Irradiated Bowel Volume in Pelvic Radiotherapy for Rectal Cancer Patients: Implications for the Radiotherapy-Related Intestinal Toxicity

**DOI:** 10.3389/fonc.2022.843704

**Published:** 2022-02-23

**Authors:** Gang Wang, Wenling Wang, Haijie Jin, Hongmin Dong, Weiwei Chen, Xiaokai Li, Saixi Bai, Guodong Li, Wanghua Chen, Leilei Li, Juan Chen

**Affiliations:** Department of Abdominal Oncology, The Affiliated Hospital of Guizhou Medical University, The Affiliated Cancer Hospital of Guizhou Medical University, Guiyang, China

**Keywords:** rectal cancer, pelvic radiotherapy, abdominal circumference, irradiated bowel volume, intestinal toxicity

## Abstract

**Background:**

To effectively reduce the irradiated bowel volume so as to reduce intestinal toxicity from pelvic radiotherapy, treatment in the prone position with a full bladder on a belly board is widely used in pelvic radiotherapy for rectal cancer patients. However, the clinical applicable condition of this radiotherapy mode is unclear. The aim of this study was to preliminarily identify patients who were not eligible for this radiotherapy mode by analyzing the effect of abdominal circumference on the irradiated bowel volume.

**Methods:**

From May 2014 to September 2019, 179 patients with locally advanced rectal cancer were retrospectively reviewed in our center. All patients received pelvic radiotherapy. Weight, height, AC, and body mass index (BMI) were used as the research objects, and the irradiated bowel volume at different dose levels (V10, V20, V30, V40, V50) was selected as the outcome variable. Multivariate linear regression and sensitivity analyses were used to evaluate the correlation between AC and irradiated bowel volume. Generalized additive model (GAM) and piecewise linear regression were used to further analyze the possible nonlinear relationship between them.

**Results:**

Among the four body size indicators, AC showed a negative linear correlation with the irradiated bowel volume, which was the most significant and stable. In adjuvant radiotherapy patients, we further discovered the threshold effect between AC and irradiated bowel volume, as AC was greater than the inflection point (about 71 cm), irradiated bowel volume decreased rapidly with the increase in AC. *t*-test showed that in patients with small AC (<71 cm), the irradiated bowel volume was significantly higher than that of patients with medium-large AC (≥71 cm). Especially in patients with adjuvant radiotherapy, the mean irradiated bowel volume of patients with small AC was the highest in this study. Compared with adjuvant radiotherapy, in neoadjuvant radiotherapy, the mean difference of irradiated bowel volume between patients with medium-large AC and those with small AC was larger.

**Conclusion:**

AC is an independent factor influencing the irradiated bowel volume and has a strong negative linear correlation with it. Patients with small AC may not benefit from this common mode of radiotherapy, especially in adjuvant radiotherapy.

## Introduction

Historically, preoperative or postoperative chemoradiotherapy has proved to improve local control and survival for locally advanced rectal cancer (1–3). Palliative radiotherapy significantly could relieve local symptoms for unresectable rectal cancer (4–6). Therefore, pelvic radiotherapy is widely utilized for locally advanced or metastatic rectal cancer (7). The intestinal toxicity (diarrhea, fecal incontinence, and late small bowel obstruction) caused by radiotherapy is the most common adverse reaction of all malignant tumors receiving pelvic radiotherapy. The overall incidence of acute and chronic bowel complications after pelvic irradiation to a dose of 50 Gy is in the order of 2%–9% (8). In rectal cancer patients receiving concurrent chemoradiotherapy, the bowel complication rate can reach as much as 25% (9).

**Figure 1:**
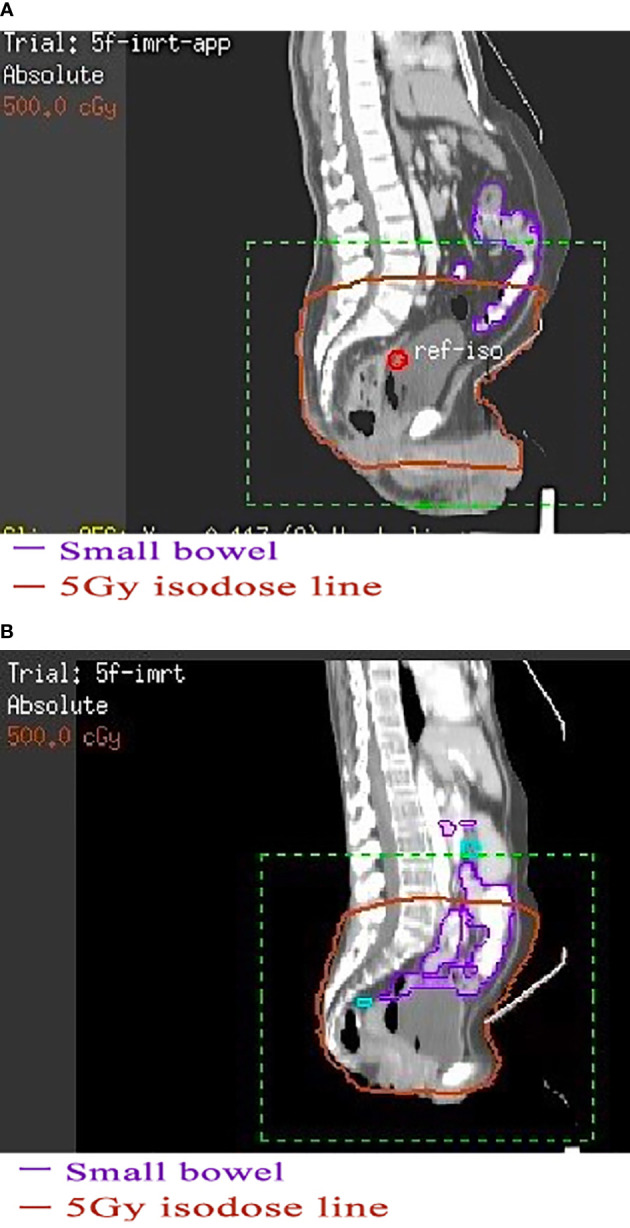
The effect of using belly board in patients with different abdominal circumferences. **(A)** Patient with a large abdominal circumference. **(B)** Patient with a small abdominal circumference. **(A)** The bowel and anterior abdominal wall in a patient with a large abdominal circumference fall well into the hollow area of the belly board. **(B)** The bowel in a patient with a small abdominal circumference hardly falls into the hollow area.

A series of clinical studies confirmed that the irradiated bowel volume was closely related to the toxicity caused by pelvic radiotherapy (9–13). Therefore, reducing the radiation dose and volume of organs at risk as much as possible is an effective measure to control the toxicity of radiotherapy and it is particularly important to achieve a low dose to the organs at risk when prescribing dose-escalated radiotherapy with sequential or simultaneous integrated boost (14, 15). For rectal cancer patients with pelvic radiotherapy, treatment in the prone position on a belly board with a full bladder was widely used to reduce the irradiated bowel volume so as to reduce the intestinal toxicity (16). Typically, this radiotherapy mode could effectively reduce the irradiated bowel volume by pushing bowel away from the irradiated region. However, in many years of clinical practice, we found that some patients with thin figure seem to be unsuitable for this mode. From these patients’ treatment-planning CT images, we found that the bowel usually did not fall well into the hollow area of the belly board (**Figures 1A, B**), and thus could not achieve the expected goal of effectively reducing irradiated bowel volume. To investigate the issue in more detail, we performed this retrospective study to determine whether the irradiated bowel volume was significantly affected by abdominal circumference (AC) of patients and to preliminarily explore whether patients with small AC are suitable for this widely used clinical radiotherapy mode, providing clinical basis for further finding a suitable mode for these patients.

## Methods and Materials

### Patient Selection

We retrospectively reviewed 190 patients with locally advanced rectal cancer who received the neoadjuvant or adjuvant radiotherapy from May 2014 to September 2019 in our center. All 190 patients used the same belly board with an inner diameter of 39 * 29 cm and was purchased from Klarity Medical & Equipment Co. Ltd (Guangzhou, China). Radiotherapy was performed in prone position on a belly board with a full bladder in all patients. Five patients underwent previous abdominal surgery, three patients chose the supine position for positioning, and another three patients’ radiotherapy plans cannot be retrieved. Therefore, 179 cases were eligible for analysis, including 67 cases of neoadjuvant radiotherapy and 112 cases of adjuvant radiotherapy.

### Treatment

In this study, the prescribed dose of planning target volume (PTV) and gross tumor volume (GTV) for neoadjuvant radiotherapy were 45 Gy at 1.8 Gy/fraction and 50 Gy at 2 Gy/fraction, respectively. The prescribed dose of PTV for adjuvant radiotherapy was 50.4 Gy at 1.8 Gy/fraction. For fair comparison, all rectal cancer target volume and organs at risk were manually contoured by the senior doctors of our center according to the same international consensus guidelines (17). Bowel volume includes the volume of small bowel and colon within 3 cm above PTV. All radiotherapy plans used 5-field intensity-modulated radiotherapy (IMRT) with radiation field angles of 0°, 45°, 95°, 265°, and 315°. Radiotherapy was performed with Elekta linear accelerator and 6MV-X. According to RTOG0822, the target dose distribution requirements are as follows: the volume of PTV receiving ≥110% prescription dose should be ≤5%. The volume of PTV receiving ≥107% prescription dose should be ≤10%. The maximum dose within PTV should be <115% of the prescribed dose. The minimum dose within PTV should be ≥93% of the prescribed dose. High dose cannot be distributed on the anastomosis, small bowel, and anal canal.

### Statistical Analysis

Four body size indicators, including weight, height, AC, and body mass index (BMI), served as subjects in our study. The irradiated bowel volume at different dose levels were used as the outcome variable, which were the bowel volume receiving more than 10 Gy (V10), 20 Gy (V20), 30 Gy (V30), 40 Gy (V40), and 50 Gy (V50). Age, gender, lesion location, T stage, N stage, PTV volume, bowel volume, and bladder volume were entered into the analysis as covariates. Multivariate linear regression analysis was performed including all variables that were significant (*p* ≤ 0.1) in the univariate analysis and variables that the researchers considered should be included. A sensitivity analysis based on multivariate linear regression was performed to assess the stability of the effects of each body size indicator on the irradiated bowel volume. To detect potential nonlinear relationships and thresholds between AC and the irradiated bowel volume, we used a flexible approach, relying on a generalized additive model (GAM) and piecewise linear regression. Means were compared by *t*-test. Statistical analyses were performed with the statistical software packages R (http://www.r-project.org, The R Foundation) and EmpowerStats (http://www.empowerstats.com, X&Y Solutions, Inc., Boston, MA, USA). *p*-values less than 0.05 (two-sided) were considered statistically significant.

## Results

The distribution of related study factors in the two groups is shown in **Table 1**. Univariate linear regression analysis was performed between 12 related variables and each irradiated bowel volume (**Supplementary Tables S1**, **S2**). Univariate linear regression showed that AC, BMI, PTV, and bowel volume had a significant linear relationship with irradiated bowel volume, while age, sex, weight, N stage, and bladder volume had a significant linear relationship with irradiated bowel volume under different conditions.

**Table 1:** The distribution of related study factors in the two groups.

	Neoadjuvant radiotherapy	Adjuvant radiotherapy
	*N* (%) or mean ± SD	*N* (%) or mean ± SD
**Number**	67	112
**Age**	54.0 ± 12.2	52.5 ± 12.1
**Gender**		
Female	15 (22.4%)	43 (38.4%)
Male	52 (77.6%)	69 (61.6%)
**Height (cm)**	162.5 ± 7.0	161.8 ± 7.7
**Weight (kg)**	56.8 ± 8.8	57.2 ± 8.9
**AC (cm)**	79.4 ± 16.1	79.2 ± 12.1
<71 cm	27 (40.3%)	39 (34.8%)
≥71 cm	40 (59.7%)	73 (65.2%)
**BMI (kg/m^2^)**	21.5 ± 2.9	21.8 ± 2.6
**Tumor location**		
Lower	39 (58.2%)	67 (59.8%)
Middle	23 (34.3%)	32 (28.6%)
Upper	5 (7.5%)	13 (11.6%)
**cT-stage**		
2	1 (1.5%)	7 (6.2%)
3	28 (41.8%)	71 (63.4%)
4	38 (56.7%)	34 (30.4%)
**cN-stage**		
0	6 (9.0%)	26 (23.2%)
1	10 (14.9%)	48 (42.9%)
2	21 (31.3%)	36 (32.1%)
*x*	30 (44.8%)	2 (1.8%)
**PTV (cm^3^)**	1,413.6 ± 345.9	1,393.5 ± 263.9
**Bowel volume (cm^3^)**	932.4 ± 346.8	967.0 ± 277.1
**Bladder volume (cm^3^)**	352.6 ± 214.7	433.5 ± 184.5
**Irradiated bowel volume (cm^3^)**		
V10	701.0 ± 305.8	753.6 ± 260.3
V20	323.5 ± 184.2	393.3 ± 182.4
V30	153.9 ± 105.1	216.1 ± 116.2
V40	93.7 ± 75.3	136.7 ± 80.9
V50	–	86.6 ± 61.5

AC, abdominal circumference; BMI, Body Mass Index; PTV, planning target volume; V10, irradiated bowel volume receiving more than 10 Gy; V20, irradiated bowel volume receiving more than 20 Gy; V30, irradiated bowel volume receiving more than 30 Gy; V40, irradiated bowel volume receiving more than 40 Gy; V50, irradiated bowel volume receiving more than 50 Gy.

Based on the results of univariate analysis and the researcher’s experience, 8 factors, namely, age, gender, height, weight, AC, N stage, PTV, and bladder volume were included as covariates in the subsequent multivariate linear regression analysis. Taking AC as an independent variable, after adjustment for relevant covariates (age, gender, height, weight, N stage, PTV, bladder volume), multivariate linear regression analysis showed a stable and strong negative linear correlation between AC and each irradiated bowel volume (**Table 2**). Unlike AC, sensitivity analysis based on multivariate linear regression indicated that the association between three body size indicators (weight, height, BMI) and each irradiated bowel volume lacked consistency and stability (**Supplementary Tables S3**–**S5**).

**Table 2:** Sensitivity analysis of AC related to each irradiated bowel volume based on multivariate linear regression.

Model	Neoadjuvant radiotherapy		Adjuvant radiotherapy	
	*β* (95% CI)	*p*	*β* (95% CI)	*p*
**V10 (cm^3^)**				
Nonadjusted	−6.4 (−10.8, −2.1)	0.005	−5.4 (−8.7, -2.1)	0.002
Adjust I	−9.8 (−14.1, −5.5)	<0.001	−5.7 (−8.6, −2.8)	<0.001
Adjust II	−11.6 (−17.0, −6.2)	<0.001	−5.3 (−9.6, −1.0)	0.017
**V20**				
Nonadjusted	−4.7 (−7.2, −2.1)	<0.001	−5.3 (−7.5, −3.0)	<0.001
Adjust I	−6.1 (−8.4, −3.7)	<0.001	−5.6 (−7.4, −3.8)	<0.001
Adjust II	−6.1 (−9.0, −3.1)	<0.001	−5.6 (−8.2, −3.1)	<0.001
**V30**				
Nonadjusted	−2.2 (−3.7, −0.7)	0.006	−2.9 (−4.4, −1.5)	<0.001
Adjust I	−2.9 (−4.2, −1.7)	<0.001	−3.1 (−4.3, −1.9)	<0.001
Adjust II	−3.1 (−4.7, −1.4)	<0.001	−3.1 (−4.8, −1.4)	<0.001
**V40**				
Nonadjusted	−1.4 (−2.5, −0.3)	0.012	−1.9 (−2.9, −0.9)	<0.001
Adjust I	−1.9 (−2.9, −0.8)	<0.001	−2.0 (−2.9, −1.1)	<0.001
Adjust II	−1.9 (−3.1, −0.6)	0.005	−2.3 (−3.6, −1.1)	<0.001
**V50**				
Nonadjusted	–		−1.6 (−2.4, −0.8)	<0.001
Adjust I	–		−1.6 (−2.3, −0.9)	<0.001
Adjust II	–		−1.5 (−3.0, −1.0)	<0.001

Exposure variable: abdominal circumference (AC); outcome variables: V10, V20, V30, V40, and V50; adjust I model adjust for: age, gender, cN-stage, PTV, and bladder volume; adjust II model adjust for: age, gender, cN-stage, PTV, bladder volume, height, and weight.

**Figure 2:**
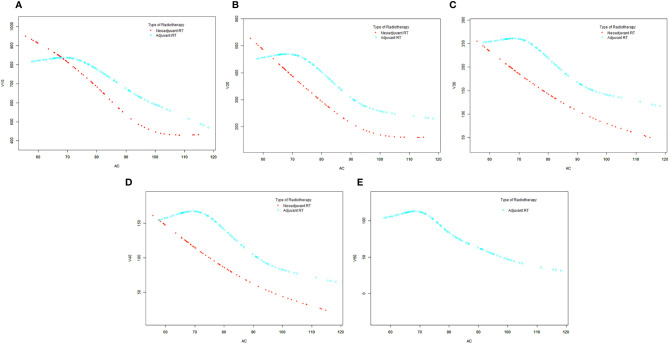
The smooth curve fits between the AC and each irradiated bowel volume base on generalized additive model (GAM). **(A)** V10. **(B)** V20. **(C)** V30. **(D)** V40. **(E)** V50. AC, abdominal circumference; V10, irradiated bowel volume receiving more than 10 Gy; V20, irradiated bowel volume receiving more than 20 Gy; V30, irradiated bowel volume receiving more than 30 Gy; V40, irradiated bowel volume receiving more than 40 Gy; V50, irradiated bowel volume receiving more than 50 Gy; RT, radiotherapy.

Generalized additive model analysis showed that there was a significant and consistent nonlinear relationship between AC and each irradiated bowel volume in the patients with adjuvant radiotherapy, but no similar relationship was found in the neoadjuvant patients (**Figure 2A**–**E**). We compared linear regression model and two-piecewise linear regression model, and the *p* for log likelihood ratio test is less than 0.05 (**Table 3**). This result indicates that the two-piecewise linear regression model should be used to fit the relationship between AC and irradiated bowel volume. By two-piecewise linear regression model and recursive algorithm, we calculated that the inflection points of AC were 70, 68, 70, 71, and 70 cm respectively to different irradiated bowel volumes (**Table 3**). On the left of the inflection point, although statistical significance was not reached, a trend was observed that all of the irradiated bowel volume increased with the increase of AC. On the right of the inflection point, all of the irradiated bowel volume decreased with the significant increase of AC and the *p*-values were less 0.05.

**Table 3:** Piecewise linear regression analysis of the AC associated with each irradiated bowel volume in adjuvant radiotherapy.

	V10 (cm^3^)	V20	V30	V40	V50
	*β* (95% CI)	*β* (95% CI)	*β* (95% CI)	*β* (95% CI)	*β* (95% CI)
**Standard linear regression**	−5.3 (−9.6, −1.0)	−5.6 (−8.2, −3.1)	−3.1 (−4.8, −1.4)	−2.3 (−3.6, −1.1)	−2.0 (−3.0, −1.0)
**Piecewise linear regression**					
Inflection points of AC (cm)	70	68	70	71	70
<Inflection point	14.1 (−0.8, 29.1)	9.3 (−2.4, 21.0)	3.7 (−2.3, 9.6)	3.0 (−1.0, 7.0)	1.4 (−2.1, 4.9)
>Inflection point	−7.9 (−12.5, −3.3)	−6.7 (−9.3, −4.1)	−4.1 (−5.9, −2.2)	−3.2 (−4.6, −1.9)	−2.5 (−3.6, −1.4)
***p* for Log-likelihood ratio test**	0.006	0.008	0.015	0.004	0.036

Exposure variable: abdominal circumference (AC); outcome variables: V10, V20, V30, V40, and V50; adjust variables: age, gender, cN-stage, PTV, bladder volume, height, and weight.

Taking 71 cm of AC (ranging from 58 to 118 cm) as the cut-off value, the irradiated bowel volume means of patients with AC <71 cm were significantly greater than that of patients with AC ≥71 cm at different dose levels (**Table 4**).

**Table 4:** The *t*-test results with regard to the irradiated bowel volume of different AC patients.

	AC		Mean difference	CI (95%)		*p* ^*^
	<71 cm	≥71 cm		Lower	Upper	
V10 (cm^3^)						
**NRT**	817.77	622.21	195.57	50.12	341.01	0.0092
**ART**	831.74	711.80	119.94	19.69	220.18	0.0195
V20						
**NRT**	410.09	265.00	145.09	60.02	230.16	0.0011
**ART**	469.82	352.43	117.40	48.87	185.92	0.0010
V30						
**NRT**	194.25	126.70	67.56	17.58	117.53	0.0088
**ART**	257.41	194.05	63.36	19.07	107.65	0.0055
V40						
**NRT**	119.22	76.49	42.72	6.49	78.96	0.0216
**ART**	161.28	123.69	37.68	6.54	68.82	0.0182
V50						
**ART**	112.80	72.56	40.20	17.12	63.27	0.0008

AC, abdominal circumference; NRT, neoadjuvant radiotherapy; ART, adjuvant radiotherapy. ^*^p-values for two-sided two-sample t-test.

## Discussion

How to effectively reduce the toxicity of radiotherapy has always been one of the hot issues in radiotherapy research. In the past, many studies compared different modes of pelvic radiotherapy for rectal cancer, in which factors compared mainly include the filling state of bladder, different body positions, and whether to combine the belly board, etc. Finally, multiple studies (18–22) have confirmed that the use of prone position with a full bladder on a belly board can significantly reduce the irradiated bowel volume at each dose level in rectal cancer patients with pelvic radiotherapy, so as to reduce intestinal toxicity caused by radiotherapy as much as possible. However, so far, few researchers have paid attention to the clinical applicable conditions of this radiotherapy mode. Our study results not only validate previous clinical observations but also provide essential information for applicable conditions to this radiotherapy mode.

This study demonstrates for the first time that under the commonly used radiotherapy mode, the AC of patients with rectal cancer had a significant negative linear correlation with irradiated bowel volume. This negative linear relationship indicates that rectal cancer patients with small AC had a congenital disadvantage in controlling the irradiated bowel volume when receiving pelvic radiotherapy in prone position on a belly board with a full bladder. The main reason for this disadvantage is that the bowel of patients with small AC cannot fall well into the hollow area of the belly board, so that more bowels remain in the pelvic cavity. In this case, no matter how to optimize the radiotherapy plan, the irradiated bowel volume cannot be effectively reduced. In rectal cancer patients who received adjuvant radiotherapy, we further discovered the threshold effect between AC and irradiated bowel volume. By two-piecewise linear regression model and recursive algorithm, we found that when the patient’s AC was greater than the value of the inflection points (about 71 cm), the patient’s irradiated bowel volume decreased rapidly with the increase in AC. This finding indicates that in adjuvant radiotherapy patients, the patient’s irradiated bowel volume can only benefit from this radiotherapy mode when the AC is greater than 71 cm. However, it is regrettable that this threshold effect could not be detected in neoadjuvant radiotherapy patients.

Based on the above findings, we took AC of 71 cm as the cutoff point (range from 58 to 118 cm) and defined AC <71 cm as small AC and AC ≥71 cm as medium-large AC. *t*-test results confirmed that irradiated bowel volume at all dose levels was significantly higher in patients with small AC than in patients with medium-large AC, and the significance of this difference was not affected by the type of radiotherapy (**Table 4**).

This study is retrospective, so no correlation analysis between intestinal toxicity and AC was performed. However, multiple previous clinical studies have confirmed that the irradiated bowel volume is significantly related to the radiotherapy-induced intestinal toxicity. Sini et al. (23) found that bowel dose volumes V10, V20, V30, V40, and V50 were significantly related to intestinal toxicity, with OR (95% CI) being 1.001 (1–1.003), 1.002 (1–1.006), 1.003 (1–1.006), 1.007 (1.002–1.012), and 1.012 (1.001–1.022), and that V20 ≤470 cm^3^, V30 ≤245 cm^3^, and V42 ≤110 cm^3^ could significantly reduce the risk of intestinal toxicity by radiotherapy. Another study of cervical cancer patients with concurrent chemoradiotherapy by Simpson et al. (24) demonstrated that the incidence of grade 2 gastrointestinal toxicities was 65% and 33% for V45 >150 and ≤150 cm^3^, respectively, and the risk of gastrointestinal toxicities was reduced by approximately 50% for every 100 cm^3^ reduction in V45. In this study, V20, V30, and V40 of patients with small AC (<71 cm) who were treated with adjuvant radiotherapy were 470, 257, and 161 cm^3^, respectively, which all reached or exceeded the threshold value that could significantly reduce the risk of intestinal toxicity reported by Sini et al. Therefore, we have reason to believe that when using this mode of radiotherapy, patients with small AC who receive adjuvant radiotherapy may have the highest risk of intestinal toxicity. For neoadjuvant radiotherapy patients, although means of V10, V20, V30, and V40 are smaller than those of adjuvant radiotherapy patients, the mean differences of V10, V20, V30, and V40 between the small AC and the medium-large AC are 196, 145, 68, and 43 cm^3^ are greater than the corresponding values of adjuvant radiotherapy patients (**Table 4**). This result indicates that compared with adjuvant radiotherapy, patients with medium-large AC can benefit more from this radiotherapy mode in neoadjuvant radiotherapy. If the OR values (1.001, 1.002, 1.003, 1.007) in the study by Sini et al. were used, compared with patients with medium-large AC, the risk of intestinal toxicity in patients with small AC may increase by 20% to 30%.

In previous studies, Kim et al. reported that with belly board the irradiated bowel volume could significantly decrease when the BMI ranges from 20 to 25, however, when the BMI was outside the above range, this advantage of using belly board did not reach statistical significance (19). Kundapur reanalyzed the original data of Baglan et al. (9) and Robertson et al. (11), and the results showed that the most benefit was provided for patients with BMI >23 in terms of irradiated bowel volume (25). Different from the previous two studies, this study conducted multivariate sensitivity analysis for more different body size indicators, and finally found that AC and irradiated bowel volume were the most closely and stably correlated, even when compared with BMI. Multivariate sensitivity analysis for BMI showed that after AC was adjusted, BMI and irradiated bowel volume no longer had significant correlation (**Supplementary Table 5**). This result also indicated that AC had a more direct impact on irradiated bowel volume than BMI.

Taken together, our study demonstrates that AC is an effective predictor of irradiated bowel volume for rectal cancer patients who used prone position on a belly board with a full bladder for pelvic radiotherapy. In addition, for patients with small AC, this radiotherapy mode does not achieve the expected goal—the significant reduction of the volume of intestinal exposure. The clinical significance of these results is that considering the increased risk of intestinal toxicity caused by more irradiated bowel volume, clinicians should be more cautious in the selection of radiotherapy mode for patients with small AC, especially in Asian countries where small body sizes are common. For patients with small AC, supine position combined with bladder filling mode may be a better choice. In order to confirm this hypothesis, a prospective controlled study is being conducted in our center.

## Conclusion

To reduce bowel dose volume and bladder filling, treatment in a prone position combined with belly board was widely used in pelvic radiotherapy for rectal cancer patients. In this mode, the AC of patient is an independent factor influencing the irradiated bowel volume and has a strong negative linear correlation with it. Patients with small AC may not benefit from this mode, especially in adjuvant radiotherapy. In contrast, patients with medium-large AC could benefit more from this radiotherapy mode in neoadjuvant radiotherapy.

## Data Availability Statement

The raw data supporting the conclusions of this article will be made available by the authors, without undue reservation.

## Ethics Statement

The studies involving human participants were reviewed and approved by The Ethics Committee of Guizhou Medical University Affiliated Cancer Hospital (The Affiliated Cancer Hospital of Guizhou Medical University, Guiyang, People’s Republic of China). Written informed consent for participation was not required for this study in accordance with the national legislation and the institutional requirements.

## Author Contributions

GW and WW contributed to conception and design of the study. GW, HJ, WHC, XL, SB, JC, and HD organized the database. GW performed the statistical analysis. GW wrote the first draft of the manuscript. GW, LL, and WWC wrote sections of the manuscript. All authors contributed to manuscript revision and read and approved the submitted version.

## Funding

This study was supported by the Innovation Team Major Research Project (QianJaoHe KY [2018]020) of Guizhou Provincial Education Department. The funders had no role in the study design, data collection and analysis, decision to publish, or preparation of the manuscript.

## Conflict of Interest

The authors declare that the research was conducted in the absence of any commercial or financial relationships that could be construed as a potential conflict of interest.

## Publisher’s Note

All claims expressed in this article are solely those of the authors and do not necessarily represent those of their affiliated organizations, or those of the publisher, the editors and the reviewers. Any product that may be evaluated in this article, or claim that may be made by its manufacturer, is not guaranteed or endorsed by the publisher.

## Abbreviations

AC, abdominal circumference; ACSVU, abdominal cross-sectional volume at the umbilical level; BMI, body mass index; GAM, generalized additive model; PTV, planning target volume; GTV, gross tumor volume; Gy, Gray; RTOG, radiation therapy oncology group; CT, computed tomography.
