# Supplementary material for: Effect of Abdominal Circumference on the Irradiated Bowel Volume in Pelvic Radiotherapy for Rectal Cancer Patients: Implications for the Radiotherapy-Related Intestinal Toxicity
Source: Front Oncol. 2022 Feb 23;12:843704. doi: 10.3389/fonc.2022.843704 (PMC8904399; doi:10.3389/fonc.2022.843704)
Supplement: Supplementary file 1 [file Table_1.docx]

Supplementary Table 1 Univariable linear regression analysis of factors related to the irradiated bowel volume in adjuvant radiotherapy patients.

|  | V10 (cm^3^) | | V20 | | V30 | | V40 | | V50 | |
| --- | --- | --- | --- | --- | --- | --- | --- | --- | --- | --- |
|  | β (95%CI) | P | β (95%CI) | P | β (95%CI) | P | β (95%CI) | P | β (95%CI) | P |
| **Age** | -4.1 (-8.2, -0.1) | 0.047 | -3.9 (-6.7, -1.0) | 0.008 | -2.2 (-4.0, -0.4) 0.016 | 0.016 | -1.5 (-2.8, -0.2) | 0.025 | -0.7 (-1.7, 0.3) | 0.150 |
| **Gender** |  |  |  |  |  |  |  |  |  |  |
| Female | Reference |  | Reference |  | Reference |  | Reference |  | Reference |  |
| Male | -164.6 (-261.3, -67.8) | 0.001 | -91.9 (-161.6, -22.2) | 0.011 | -51.8 (-96.6, -7.0) | 0.025 | -49.0 (-80.0, -17.9) | 0.003 | -47.6 (-70.2, -24.9) | <0.001 |
| **Height（cm）** | -1.3 (-7.7, 5.0) | 0.676 | 0.4 (-4.0, 4.8) | 0.856 | 1.0 (-1.8, 3.8) | 0.485 | 0.1 (-1.8, 2.1) | 0.911 | -0.0 (-1.5, 1.4) | 0.949 |
| **Weight (Kg)** | -7.1 (-12.4, -1.7) | 0.011 | -5.4 (-9.1, -1.7) | 0.005 | -2.7 (-5.1, -0.3) | 0.027 | -1.9 (-3.5, -0.02) | 0.030 | -1.7 (-2.9, -0.4) | 0.010 |
| **AC (cm)** | -5.4 (-8.7, -2.1) | 0.002 | -5.3 (-7.5, -3.0) | <0.001 | -2.9 (-4.4, -1.5) | <0.001 | -1.9(-2.9, -0.9) | <0.001 | -1.6 (-2.4, -0.8) | <0.001 |
| **BMI** | -25.5 (-43.2, -7.7) | 0.006 | -22.7 (-34.8, -10.5) | <0.001 | -13.1 (-21.0, -5.3) | 0.001 | -7.7 (-13.2, -2.2) | 0.008 | -6.6 (-10.7, -2.4) | 0.003 |
| **Tumor Location** |  |  |  |  |  |  |  |  |  |  |
| Lower | Reference |  | Reference |  | Reference |  | Reference |  | Reference |  |
| Middle | -36.9 (-149.5, 75.7) | 0.522 | -27.2 (-106.7, 52.3) | 0.504 | -8.8 (-59.6, 41.9) | 0.734 | -4.3 (-40.1, 31.6) | 0.816 | 3.3 (-23.7, 30.2) | 0.812 |
| Upper | -14.2 (-173.0, 144.6) | 0.861 | 13.0 (-99.1, 125.2) 0 | 0.820 | 20.8 (-50.8, 92.5) | 0.570 | 15.2 (-35.4, 65.8) | 0.556 | 16.1 (-21.9, 54.0) | 0.409 |
| **cT-stage** |  |  |  |  |  |  |  |  |  |  |
| 2 | Reference |  | Reference |  | Reference |  | Reference |  | Reference |  |
| 3 | -9.6 (-217.3, 198.0) | 0.928 | -27.9 (-174.4, 118.7) | 0.710 | -6.2 (-99.6, 87.1) | 0.896 | -3.2 (-69.3, 62.8) | 0.923 | 10.9 (-38.5, 60.2) | 0.667 |
| 4 | 21.9 (-195.7, 239.4) | 0.844 | 3.9 (-149.6, 157.4) | 0.961 | 21.5 (-76.3, 119.3) | 0.667 | 12.5 (-56.7, 81.7) | 0.723 | 26.9 (-24.9, 78.6) | 0.311 |
| **cN-stage** |  |  |  |  |  |  |  |  |  |  |
| 0 | Reference |  | Reference |  | Reference |  | Reference |  | Reference |  |
| 1 | 175.9 (52.0, 299.8) | 0.006 | 141.0 (54.9, 227.1) | 0.002 | 82.5 (27.1, 137.9) | 0.004 | 58.4 (19.4, 97.5) | 0.004 | 39.8 (10.3, 69.3) | 0.010 |
| 2 | 124.7 (-6.2, 255.7) | 0.065 | 91.2 (0.2, 182.2) | 0.052 | 58.6 (0.1, 117.2) | 0.052 | 40.5 (-0.8, 81.7) | 0.057 | 30.5 (-0.7, 61.7) | 0.058 |
| x | 27.0 (-346.4, 400.4) | 0.887 | -83.2 (-342.6, 176.2) | 0.531 | -50.1 (-217.1, 116.9) | 0.557 | -44.8 (-162.5, 72.9) | 0.457 | -37.1 (-126.1, 51.8) | 0.415 |
| **PTV (cm^3^)** | 0.5 (0.3, 0.6) | <0.001 | 0.4 (0.3, 0.5) | <0.001 | 0.2 (0.2, 0.3) | <0.001 | 0.1 (0.1, 0.2) | <0.001 | 0.1 (0.0, 0.1) | <0.001 |
| **Bowel Volume (cm^3^)** | 0.8 (0.7, 0.9) | <0.001 | 0.5 (0.4, 0.6) | <0.001 | 0.3 (0.2, 0.4) | <0.001 | 0.2 (0.1, 0.2) | <0.001 | 0.1 (0.1, 0.2) | <0.001 |
| **Bladder Volume (cm^3^)** | -0.1 (-0.4, 0.1) | 0.382 | -0.1 (-0.3, 0.1) | 0.244 | -0.1 (-0.2, 0.1) | 0.342 | -0.1 (-0.1, 0.0) | 0.256 | 0.0 (-0.1, 0.1) | 0.851 |

AC: Abdominal Circumference; BMI: Body Mass Index; PTV: Planning Target Volume;

Supplementary Table 2 Univariable linear regression analysis of factors related to the irradiated bowel volume in neoadjuvant radiotherapy patients.

|  | V10 (cm^3^) | | V20 | | V30 | | V40 | |
| --- | --- | --- | --- | --- | --- | --- | --- | --- |
|  | β (95%CI) | P | β (95%CI) | P | β (95%CI) | P | β (95%CI) | P |
| **Age** | 3.5 (-2.8, 9.8) | 0.282 | 0.5 (-3.2, 4.2) | 0.783 | 0.0 (-2.1, 2.1) | 0.982 | 0.1 (-1.4, 1.6) | 0.896 |
| **Gender** |  |  |  |  |  |  |  |  |
| Female | Reference |  | Reference |  | Reference |  | Reference |  |
| Male | -117.5 (-299.3, 64.3) | 0.210 | -15.0 (-121.5, 91.6) | 0.784 | 16.3 (-44.4, 77.1) | 0.600 | 11.0 (-32.5, 54.5) | 0.621 |
| **Height（cm）** | -3.9 (-14.6, 6.7) | 0.471 | 1.2 (-5.2, 7.6) | 0.716 | 1.2 (-2.5, 4.8) | 0.531 | 1.1 (-1.5, 3.8) | 0.399 |
| **Weight (Kg)** | -8.7 (-16.9, -0.5) | 0.041 | -6.5 (-11.3, -1.6) | 0.011 | -2.7 (-5.6, 0.1) | 0.062 | -1.8 (-3.8, 0.2) | 0.089 |
| **AC (cm)** | -6.4 (-10.8, -2.61 | 0.005 | -4.7 (-7.2, -2.1) | <0.001 | -2.2 (-3.7, -0.7) | 0.006 | -1.4 (-2.5, -0.3) | 0.012 |
| **BMI** | -24.4 (-49.1, -0.3) | 0.058 | -23.8 (-38.0, -9.6) | 0.002 | -10.9 (-19.3, -2.6) | 0.012 | -7.7 (-13.6, -1.7) | 0.015 |
| **Tumor Location** |  |  |  |  |  |  |  |  |
| Lower | Reference |  | Reference |  | Reference |  | Reference |  |
| Middle | 71.3 (-94.1, 236.7) | 0.401 | 35.8 (-59.5, 131.0) | 0.464 | 12.4 (-42.1, 66.8) | 0.658 | 4.0 (-35.4, 43.3) | 0.844 |
| Upper | 52.2 (-246.7, 351.1) | 0.733 | 100.3 (-71.8, 272.4) | 0.258 | 57.8 (-40.6, 156.1) | 0.254 | 16.7 (-54.4, 87.8) | 0.647 |
| **cT-stage** |  |  |  |  |  |  |  |  |
| 2 | Reference |  | Reference |  | Reference |  | Reference |  |
| 3 | 428.5 (-202.2, 1059.1) | 0.188 | 162.6 (-198.9, 524.0) | 0.381 | 35.1 (-171.8, 242.1) | 0.741 | 7.7 (-142.3, 157.7) | 0.920 |
| 4 | 490.2 (-137.6, 1118.0) | 0.131 | 239.8 (-120.0, 599.6) | 0.196 | 82.9 (-123.1, 288.9) | 0.433 | 34.8 (-114.5, 184.1) | 0.649 |
| **cN-stage** |  |  |  |  |  |  |  |  |
| 0 | Reference |  | Reference |  | Reference |  | Reference |  |
| 1 | 8.6 (-301.0, 318.1) | 0.957 | 40.0 (-129.7, 209.7) | 0.646 | 21.5 (-73.4, 116.4) | 0.659 | 16.8 (-52.8, 86.4) | 0.638 |
| 2 | -4.4 (-282.0, 273.1) | 0.975 | 24.9 (-127.2, 177.0) | 0.749 | -9.1 (-94.2, 75.9) | 0.834 | -3.1 (-65.5, 59.3) | 0.923 |
| x | 216.3 (-51.8, 484.4) | 0.119 | 192.1 (45.1, 339.1) | 0.013 | 101.8 (19.6, 184.0) | 0.018 | 69.5 (9.3, 129.8) | 0.027 |
| **PTV (cm^3^)** | 0.3 (0.1, 0.6) | 0.002 | 0.2 (0.1, 0.3) | 0.001 | 0.1 (0.1, 0.2) | <0.001 | 0.1 (0.0, 0.1) | 0.001 |
| **Bowel Volume (cm^3^)** | 0.9 (0.8, 0.9) | <0.001 | 0.4 (0.4, 0.5) | <0.001 | 0.2 (0.2, 0.3) | <0.001 | 0.1 (0.1, 0.2) | <0.001 |
| **Bladder Volume (cm^3^)** | -0.4 (-0.7, -0.0) | 0.053 | -0.3 (-0.5, -0.1) | 0.002 | -0.2 (-0.3, -0.1) | <0.001 | -0.2 (-0.2, -0.1) | <0.001 |

AC: Abdominal Circumference; BMI: Body Mass Index; PTV: Planning Target Volume.

Supplementary Table 3 Sensitivity analysis of **weight** related to each irradiated bowel volume based on multivariate linear regression.

|  | **Neoadjuvant radiotherapy** | | **Adjuvant radiotherapy** | |
| --- | --- | --- | --- | --- |
|  | **β (95%CI)** | **P** | **β (95%CI)** | **P** |
| **V10** |  |  |  |  |
| Non-adjusted | -8.7 (-16.9, -0.5) | 0.041 | -7.1 (-12.4, -1.7) | 0.011 |
| Adjust I | -6.9 (-15.8, 1.9) | 0.130 | -5.8 (-11.0, -0.6) | 0.030 |
| Adjust II | 6.3 (-4.4, 17.0) | 0.256 | -2.3 (-10.7, 6.1) | 0.598 |
| **V20** |  |  |  |  |
| Non-adjusted | -6.5 (-11.3, -1.6) | 0.011 | -5.4 (-9.1, -1.7) | 0.005 |
| Adjust I | -5.6 (-10.5, -0.7) | 0.028 | -5.5 (-8.9, -2.1) | 0.002 |
| Adjust II | -0.2 (-6.1, 5.7) | 0.953 | -1.0 (-6.0, 4.0) | 0.692 |
| **V30** |  |  |  |  |
| Non-adjusted | -2.7 (-5.6, 0.1) | 0.062 | -2.7 (-5.1, -0.3) | 0.027 |
| Adjust I | -2.6 (-5.3, -0.0) | 0.053 | -2.7 (-4.9, -0.5) | 0.019 |
| Adjust II | 0.3 (-2.9, 3.5) | 0.850 | -0.8 (-4.1, 2.6) | 0.655 |
| **V40** |  |  |  |  |
| Non-adjusted | -1.8 (-3.8, 0.2) | 0.089 | -1.9 (-3.5, -0.2) | 0.030 |
| Adjust I | -1.6 (-3.6, 0.4) | 0.117 | -1.4 (-3.0, 0.2) | 0.095 |
| Adjust II | -0.0 (-2.6, 2.5) | 0.974 | 0.4 (-2.1, 2.9) | 0.752 |
| **V50** |  |  |  |  |
| Non-adjusted | ~~-------------------~~ |  | -1.7 (-2.9, -0.4) | 0.010 |
| Adjust I | ~~-------------------~~ |  | -1.0 (-2.3, 0.3) | 0.145 |
| Adjust II | ~~-------------------~~ |  | 0.6 (-1.4, 2.5) | 0.576 |

Exposure variable: weight;
Outcome variables: V10, V20, V30, V40, V50;
Adjust I model adjust for: age, gender, cN-stage, PTV, bladder volume;

Adjust II model adjust for: age, gender, cN-stage, PTV, bladder volume, height, AC.

Supplementary Table 4 Sensitivity analysis of **height** related to each irradiated bowel volume based on multivariate linear regression.

|  | **Neoadjuvant radiotherapy** | | **Adjuvant radiotherapy** | |
| --- | --- | --- | --- | --- |
|  | **β (95%CI)** | **P** | **β (95%CI)** | **P** |
| **V10** |  |  |  |  |
| Non-adjusted | -3.9 (-14.6, 6.7) | 0.471 | -1.3 (-7.7, 5.0) | 0.676 |
| Adjust I | -3.5 (-15.5, 8.6) | 0.572 | 3.9 (-3.0, 10.8) | 0.271 |
| Adjust II | -4.7 (-16.6, 7.2) | 0.440 | 7.4 (-0.5, 15.3) | 0.070 |
| **V20** |  |  |  |  |
| Non-adjusted | 1.2 (-5.2, 7.6) | 0.716 | 0.4 (-4.0, 4.8) | 0.856 |
| Adjust I | 0.7 (-6.1, 7.5) | 0.843 | 3.3 (-1.3, 7.9) | 0.165 |
| Adjust II | 2.1 (-4.5, 8.6) | 0.540 | 6.1 (1.3, 10.8) | 0.014 |
| **V30** |  |  |  |  |
| Non-adjusted | 1.2 (-2.5, 4.8) | 0.531 | 1.0 (-1.8, 3.8) | 0.485 |
| Adjust I | -0.1 (-3.7, 3.5) | 0.972 | 3.2 (0.2, 6.1) | 0.037 |
| Adjust II | 0.4 (-3.2, 4.0) | 0.833 | 4.8 (1.7, 7.9) | 0.003 |
| **V40** |  |  |  |  |
| Non-adjusted | 1.1 (-1.5, 3.8) | 0.399 | 0.1 (-1.8, 2.1) | 0.911 |
| Adjust I | 0.6 (-2.1, 3.3) | 0.675 | 2.1 (0.0, 4.2) | 0.049 |
| Adjust II | 1.0 (-1.8, 3.8) | 0.491 | 2.7 (0.4, 5.1) | 0.022 |
| **V50** |  |  |  |  |
| Non-adjusted | ~~-------------------~~ |  | -0.0 (-1.5, 1.4) | 0.949 |
| Adjust I | ~~-------------------~~ |  | 2.0 (0.3, 3.7) | 0.021 |
| Adjust II | ~~-------------------~~ |  | 2.4 (0.5, 4.2) | 0.012 |

Exposure variable: height;
Outcome variables: V10, V20, V30, V40, V50;
Adjust I model adjust for: age, gender, cN-stage, PTV, bladder volume;

Adjust II model adjust for: age, gender, cN-stage, PTV, bladder volume, weight, AC.

Supplementary Table 5 Sensitivity analysis of **BMI** related to each irradiated bowel volume based on multivariate linear regression.

|  | **Neoadjuvant radiotherapy** | | **Adjuvant radiotherapy** | |
| --- | --- | --- | --- | --- |
|  | **β (95%CI)** | **P** | **β (95%CI)** | **P** |
| **V10** |  |  |  |  |
| Non-adjusted | -24.4 (-49.1, 0.4) | 0.058 | -25.5 (-43.2, -7.7) | 0.006 |
| Adjust I | -19.4 (-45.8, 7.0) | 0.156 | -24.2 (-39.6, -8.9) | 0.003 |
| Adjust II | 16.7 (-11.7, 45.1) | 0.254 | -6.3 (-28.0, 15.4) | 0.570 |
| **V20** |  |  |  |  |
| Non-adjusted | -23.8 (-38.0, -9.6) | 0.002 | -22.7 (-34.8, -10.5) | <0.001 |
| Adjust I | -19.8 (-34.1, -5.4) | 0.009 | -22.7 (-32.5, -12.9) | <0.001 |
| Adjust II | -1.4 (-17.0, 14.3) | 0.865 | -3.7 (-16.9, 9.6) | 0.587 |
| **V30** |  |  |  |  |
| Non-adjusted | -10.9 (-19.3, -2.6) | 0.012 | -13.1 (-21.0, -5.3) | 0.001 |
| Adjust I | -8.7 (-16.4, -1.0) | 0.031 | -13.0 (-19.5, -6.6) | <0.001 |
| Adjust II | 0.7 (-7.9, 9.2) | 0.880 | -3.1 (-12.0, 5.8) | 0.492 |
| **V40** |  |  |  |  |
| Non-adjusted | -7.7 (-13.6, -1.7) | 0.015 | -7.7 (-13.2, -2.2) | 0.008 |
| Adjust I | -6.0 (-11.9, -0.2) | 0.047 | -7.2 (-12.0, -2.5) | 0.003 |
| Adjust II | -0.4 (-7.2, 6.3) | 0.905 | 0.3 (-6.3, 6.8) | 0.935 |
| **V50** |  |  |  |  |
| Non-adjusted | ~~-------------------~~ |  | -6.6 (-10.7, -2.4) | 0.003 |
| Adjust I | ~~-------------------~~ |  | -5.7 (-9.5, -1.9) | 0.004 |
| Adjust II | ~~-------------------~~ |  | 0.6 (-4.6, 5.8) | 0.816 |

Exposure variable: BMI;
Outcome variables: V10, V20, V30, V40, V50;
Adjust I model adjust for: age, gender, cN-stage, PTV, bladder volume;

Adjust II model adjust for: age, gender, cN-stage, PTV, bladder volume, AC.
